# Elevated mRNA-Levels of Gonadotropin-Releasing Hormone and Its Receptor in Plaque-Bearing Alzheimer's Disease Transgenic Mice

**DOI:** 10.1371/journal.pone.0103607

**Published:** 2014-08-04

**Authors:** Syed Nuruddin, Gry Helen Enger Syverstad, Sveinung Lillehaug, Trygve B. Leergaard, Lars N. G. Nilsson, Erik Ropstad, Anette Krogenæs, Ira Ronit Hebold Haraldsen, Reidun Torp

**Affiliations:** 1 Norwegian School of Veterinary Science, Oslo, Norway; 2 Department of Anatomy, Institute of Basic Medical Sciences, University of Oslo, Oslo, Norway; 3 Department of Neuropsychiatry and Psychosomatic Medicine, Division of Surgery and Clinical Neuroscience, Oslo University Hospital - Rikshospitalet, Oslo, Norway; 4 Department of Pharmacology, University of Oslo and Oslo University Hospital, Oslo, Norway; 5 Department of Public Health & Caring Sciences / Geriatrics, Uppsala University, Uppsala, Sweden; John Hopkins University School of Medicine, United States of America

## Abstract

Research on Alzheimer's disease (AD) has indicated an association between hormones of the hypothalamic–pituitary–gonadal (HPG) axis and cognitive senescence, indicating that post meno-/andropausal changes in HPG axis hormones are implicated in the neuropathology of AD. Studies of transgenic mice with AD pathologies have led to improved understanding of the pathophysiological processes underlying AD. The aims of this study were to explore whether mRNA-levels of gonadotropin-releasing hormone (Gnrh) and its receptor (Gnrhr) were changed in plaque-bearing Alzheimer's disease transgenic mice and to investigate whether these levels and amyloid plaque deposition were downregulated by treatment with a gonadotropin-releasing hormone analog (Gnrh-a; Leuprorelin acetate). The study was performed on mice carrying the Arctic and Swedish amyloid-β precursor protein (AβPP) mutations (tgArcSwe). At 12 months of age, female tgArcSwe mice showed a twofold higher level of *Gnrh* mRNA and more than 1.5 higher level of *Gnrhr* mRNA than age matched controls. Male tgArcSwe mice showed the same pattern of changes, albeit more pronounced. In both sexes, Gnrh-a treatment caused significant down-regulation of *Gnrh* and *Gnrhr* mRNA expression. Immunohistochemistry combined with quantitative image analysis revealed no significant changes in the plaque load after Gnrh-a treatment in hippocampus and thalamus. However, plaque load in the cerebral cortex of treated females tended to be lower than in female vehicle-treated mice. The present study points to the involvement of hormonal changes in AD mice models and demonstrates that these changes can be effectively counteracted by pharmacological treatment. Although known to increase in normal aging, our study shows that *Gnrh/Gnrhr* mRNA expression increases much more dramatically in tgArcSwe mice. Treatment with Leuprorelin acetate successfully abolished the transgene specific effects on *Gnrh/Gnrhr* mRNA expression. The present experimental approach should serve as a platform for further studies on the usefulness of Gnrh-a treatment in suppressing plaque development in AD.

## Introduction

Alzheimer's disease (AD) is the most common form of dementia. The disease affects more than 35 million people worldwide [1] and each year 4.6 million new cases are diagnosed [2]. Although much is known about the neuropathology of AD, the etiology remains unclear and currently there is no cure for this neurodegenerative disease.

It is assumed that AD pathogenesis perturbs signal transduction pathways and that this contributes to neurodegeneration. Alterations in different neurotransmitter systems are well established, perhaps most clearly affecting cholinergic and glutamatergic pathways [3]. Misprocessing of amyloid-β precursor protein (AβPP) plays a pivotal role in inherited forms of AD [4], but oxidative, inflammatory [5] and hormonal processes [6], [7] might additionally and significantly contribute to the pathogenesis.

Hormonal mechanisms underlying AD development have gained renewed interest. It was recently reported that gonadotropin-releasing hormone (Gnrh) induces adult neurogenesis in several brain regions typically afflicted by AD neuropathology [8]. Furthermore, Gnrh agonist therapy has been shown to decelerate aging in animals [8] and reduce the risk of developing AD in prostate cancer patients [9].

Gnrh is a decapeptide hormone. There are three forms of Gnrh (1, 2 and 3), of which Gnrh1 and 2 are present in reptiles, birds and mammals. Gnrh1 and 2 are not only expressed in the hypothalamus but also in other brain regions, e.g. caudate nucleus, hippocampus and amygdala [10]–[12]. Based on the wide distribution of Gnrh, this hormone is likely to have roles beyond its endocrine function, possibly serving as a neurotransmitter or a modulator of neuroplasticity [13]–[17]. There is only one single functional Gnrh receptor (Gnrhr) in most species, including mice. Gnrhr is a unique rhodopsin-like G protein-coupled receptor that appears to mediate a wide variety of Gnrh1 and 2 signaling mechanisms [18].

Furthermore, recent research has elucidated cognitive and physiological effects of Gnrh [19]–[21]. Results from mice and human studies of both sexes indicate that modulation of Gnrh and its receptor by use of a Gnrh analog (Gnrh-a) may lead to significant changes in cognitive functions [19], [22], [23].

The hippocampal region is most vulnerable to AD and turns out to be particularly rich in Gnrh receptors [12], [18]. Therefore in this study, we have focused on Gnrh and receptor gene regulation in the hippocampus.

It is proposed that estrogen has a modifying effect on Alzheimer's disease [24]. As Gnrh is elevated post-menopause due to the loss of estrogen feedback, Gnrh may have a direct effect on neurodegeneration [12], [25]. Therefore, the aim of our study was to investigate the effect of Leuprorelin acetate, a Gnrh-analog, on *Gnrh* and *Gnrhr* mRNA-expression, as well as on amyloid-β (Aβ) deposition in transgenic mice expressing amyloid-β precursor protein (AβPP) mutations with the Arctic and Swedish mutations (tgArcSwe), an established model of AD [26], [27].

## Materials and Methods

### Animal model

In this study we used transgenic mice carrying a human AβPP cDNA with the Arctic (E693G) and Swedish (KM670/671NL) mutations. The animals were housed under standard conditions (12 h dark/light cycles) with unrestricted access to food and water, and sacrificed after 4 or 12 months. All animal procedures were in accordance with the National Institutes of Health Guide for the care and use of laboratory animals (FELASA) and approved by the Biological Research Ethics Committee in Norway (FOTS/3209).

Gnrh-a can be used to block the hypothalamic-pituitary-gonadal axis, inhibiting the production of gonadal hormones in mice as effectively as in humans. Gnrh-a initially induces a sharp increase in serum concentrations of the pituitary gonadotropins luteinizing hormone (LH) and follicle-stimulating hormone (FSH), known as the ‘flare effect’ that leads to an increase in serum sex steroids (within 3–4 days) but this is followed by down-regulation of Gnrhr resulting in suppression of gonadotropins and sex steroid secretion within 2 weeks [28]. In fact, chronic Gnrh-a treatment results in downstream signaling of intracellular pathways that are downstream of Gnrhr in the pituitary gonadotrophs. There are considerable data suggesting that activation of Gnrhr may regulate downstream signaling systems in a cell specific manner. Activation of Gnrhr signaling pathways can differ in hippocampus compared to pituitary [29].

In our study, animals (tgArcSwe and wild–type mice) were injected subcutaneously with 25 ng/g of the Gnrh-a Leuprorelin acetate (Procren Depot “AbbVie”) dissolved in physiological saline, or vehicle alone. The injections were given once every fourth week from the age of 4 months, before plaque deposition has begun [27]. Of the animals included in the study, about 20 % of the animals died of unknown causes before reaching the age of 12 months. This included both treated and untreated animals, leaving us with the number of animals referred to in Table 1. The remaining animals were sacrificed at 12 months, 2 weeks after receiving the last injection, and used for quantitative real time PCR (qRT-PCR) and immunohistochemical investigations. In addition to this, gene expression was analyzed with qRT-PCR in 4 months old animals who did not receive any pharmacological intervention. A subset of the untreated 12 months old animals were analyzed at the same time by the same people and used for anatomical characterization of the tgArcSwe model, therefore referred to as historical samples [26]. This is in line with the guidelines calling for “replacement, reduction, and refinement” in animal research and enabled us to reduce the number of animals for the present series of studies without compromising the quality of our analysis. Details are shown in Table 1.

**Table 1:** Animals involved in the study.

Age (in months)	12	12	4
Treatment *(Leuprorelin acetate; 25 ng/g)*	Treated	Untreated	Untreated
TgArcSwe males	3	6*	6
Wild-type males	-	6	6
TgArcSwe females	7	6*	6
Wild-type females	-	6	6

*A subset of these animals was also anatomically characterized in reference [26].

### Tissue processing

Animals were anesthetized using Isofluran Baxter (Isoflo, Abbot Laboratories, Abbot Park, IL, USA) and sacrificed at 4 or 12 months by decapitation. The brain was extracted, and divided into its two hemispheres and frozen using Nordfjord cool spray (Norden Olje, Ski, Norway) and dry ice. The tissue was stored at −80°C until further use. The right hemisphere was used for immunohistochemistry, while hippocampus from the left hemisphere was used for qRT-PCR.

### Blood samples and hormone assays

Approximately, 2 ml blood from each mouse were acquired by exsanguination during decapitation, and collected into a chilled tube containing ethylenediamine tetracetate (EDTA) and spun at 4000 rpm for 15 minutes to prepare serum. Serum samples were stored at −20°C until processed for estradiol (E2) and testosterone measurements. E2 were analyzed using DELFIA kit from PerkinElmer Life Sciences, Wallac Oy, Turku, Finland. Testosterone was analyzed using RIA-kit from Cis-bio, Electra-box, Diagnostica As, Norway. The detection limit of testosterone was 0,5 nmol/l.

### RNA Isolation

Total RNA was isolated from the hippocampus with TRIzol Reagent (Invitrogen, Paisley, UK). For each sample, 11-14 mg of frozen tissue was homogenized in 1 ml of Trizol reagent using MagNA Lyser Green Beads (Roche Diagnostics, Mannheim, Germany) and Retsch MM 301 mixer mill (Retsch GmbH, Haan, Germany). Purified RNA was dissolved in 50 µl of RNase-free water (Qiagen, Crawley, UK) followed by DNase I treatment (RNase-Free DNase Set, Qiagen, Crawley, UK) for 20 minutes at room temperature, immediately followed by purification using RNeasy Mini-Kit (Qiagen, Crawley, UK) according to the manufacturer's recommendations. The concentration and quality of the RNA were determined using NanoDrop (Thermo-Scientific, Waltham, MA, USA) and Agilent 2100 Bioanalyzer (Agilent Technologies, Santa Clara, CA, USA), respectively. RNA was stored at −80°C until further processing.

### Quantitative RT-PCR (qRT-PCR)

To predict the most stable reference gene under the present study condition [30], the GeNorm-method was used. GeNorm human detection kit and software was obtained from PrimerDesign Ltd (Southampton, UK). Of the 6 reference genes tested phosphoglycerate kinase 1 (*Pgk1*), ribosomal protein S18 (*Rps18*), beta-2 microglobulin (*B2m*), glyceraldehyde-3-phosphate dehydrogenase (*Gapdh*), glucuronidase, beta (*Gusb*), transferrin receptor (*Tfrc*), the two most stable genes; *B2m* and *Gusb* were selected as reference genes in this study.

Genes included in the gene expression analyses are listed in Table 2. Primer sequences for *Gnrh, Gnrhr, B2m* and *Gusb* were designed by using primer-3 plus, a web-based primer designing tool [31]. All primers were synthesized by Sigma-Aldrich (St. Louis, MO, USA). Specificities of all primer pairs were checked using nucleotide BLAST and primer BLAST (http://blast.ncbi.nlm.nih.gov/Blast.cgi).

**Table 2:** Primer sequences for target and reference genes used in the study.

Gene abbreviation	Forward primer	Reverse primer	Product length	Accession number
*Gnrh*	GCTCCAGCCAGCACTGGTCCTA	TGATCCACCTCCTTGCCCATCTCTT	100	NM_008145.2
*Gnrhr*	ATTAGCCTGGACCGCTCCCTGG	CATTGCGAGAAGACTGTGGGCCC	182	NM_010323
*B2m*	CTTCAGTCGTCAGCATGGCTCGT	TTTCTGGATAGCATACAGGCCGGC	83	NM_009735.3
*Gusb*	AAGGCGCTGGACGGACTGTGG	AGACTGGGCCCGACTCCCGTA	109	NM_010368.1

Primer annealing temperature and cDNA concentration were optimized before the experiment was carried out. All products were run on an agarose-ethidium bromide gel as to verify distinct bands of a size matching the intended product in the absence of primer-dimer formations.

cDNA synthesis and qRT-PCR were performed using superscript III platinum Two-Step qRT-PCR kit with SYBR Green (Invitrogen, Carlsbad, CA, USA) according to the protocol recommended by the manufacturer. A Peltier Thermal Cycler-225 (MJ Research, Waltham, MA, USA) was used to synthesize cDNA, and qRT-PCR was done with an ABI PRISM 7900 Sequence Detector System (PE Applied Biosystems, CA, USA). Technical duplicates of RNA samples, negative controls without reverse transcriptase as well as negative controls devoid of an RNA template all underwent cDNA synthesis. 1 µg of total RNA was used to synthesize cDNA (10 min at 25°C, 50 min at 42°C and 5min at 85°C). The reaction mixture (20 µl) contained 3 µl of cDNA (15 ng), 10 µl of Platinum SYBR Green qRT-PCR Super Mix-UDG, 0.2 µl of ROX reference dye, 1 µl of 10 µmol of forward and 1 µl of 10 µmol of reverse primers, and 4.8 µl of DNase/RNase-free water (Invitrogen). Cycling conditions were: initial denaturation at 95°C for 2 min; 40 amplification cycles were at 95°C for 30 s, 62°C for 30 s, and 72°C for 30 s. Each assay was performed in quadruplicate, and three negative controls were run for every assay: no template (sample lacking cDNA), no reverse transcriptase, and no RNA in reverse transcriptase reaction. The absence of primer-dimers, genomic DNA, and other DNA contaminations were also monitored by melting curve analysis at the end of each run (ABI PRISM 7900 manufacturer's recommended default settings).

### qRT-PCR analysis

The ΔCt was calculated from the difference in expression between the gene of interest (*Gnrh, Gnrhr and A*β*PP*) and mean expression of the two reference genes (*Gusb and B2m*). To investigate the effect of genetic modification in transgenic mice at 4 months and 12 months, the ΔΔCt was calculated by the difference between the ΔCt value of wild-type mice (WT mice) and tgArcSwe mice samples, within both sexes. In order to assess any effects of treatment at 12 months of age, the ΔΔCt was calculated as the difference between the ΔCt value of vehicle-treated tgArcSwe mice and Leuprorelin acetate treated tgArcSwe mice samples, within both sexes. Relative gene expression expressed as fold change was calculated by using 2^−ΔΔCt^.

### Immunohistochemistry

Sagittal cryosections (25 µm; Leica CM3050 S) from the right hemisphere were stored at −20°C. All sections were post–fixed with 4% formaldehyde (PFA) for 5 minutes, pretreated with 80% formic acid for 2 minutes and 2% H_2_O_2_ for 7 minutes. After washing with 10 mM phosphate buffered saline (PBS), pre - incubation solution (10% normal goat serum (NGS), 1% bovine serum albumin (BSA), 0.5% Triton X-100 in 10 mM PBS) was applied to the sections for 30 minutes at room temperature. Afterwards, the sections were incubated with an Aβx-40-specific polyclonal primary antibody (0.5 µg/ml; Agrisera, Umeå, Sweden) diluted 1∶2000 in primary antibody solution (3% NGS, 1% BSA, 0.5% Triton X-100 in 10 mM PBS) at 4°C overnight. The antisera was generated and evaluated for specificity as described [26], [32].

After washing steps, the sections were incubated for 1 hour with a biotinylated goat-anti-rabbit antibody (BA-1000, Vector Laboratories, CA, USA) diluted at 1∶300 in 3% NGS, 1% BSA, 0.5% Triton X-100 in 10 mM PBS, washed in 10 mM PBS, and afterwards, incubated 1 hour at room temperature with streptavidin-biotinylated horseradish peroxidase complex diluted at 1∶100 in 0.5% Triton X-100 in 10 mM PBS. After washing with 10 mM PBS, all sections were incubated with 3,3′-diaminobenzidine tetrahydrochloride (Sigma, MO, USA) for 5 minutes, before 0.1% H_2_O_2_ was added to 10 ml DAB solution and applied on the sections until proper labeling was achieved (3 minutes). The sections were briefly rinsed in water, mounted and stored at room temperature.

### Image acquisition and quantitative analyses

An automated slide scanner system (Mirax Scan, Carl Zeiss MicroImaging GmbH, Jena, Germany) was used for acquiring high-resolution TIFF images with a spatial resolution of 0.205 µm/pixel. Images were inspected by virtual microscopy using the Panoramic viewer software (3D Histech, Budapest, Hungary). Using the export functionality of the Mirax Viewer, images were scaled 1∶16 (to a spatial resolution of 3.28 µm/pixel). To compensate for differences in background color intensity following immunohistochemistry, all image histograms were normalized using the match-color algorithm in Adobe Photoshop CS6 with a photomicrograph of a wild-type section as reference [33]. Afterwards, quantitative image analysis was performed in three regions of interest (ROIs) using ImageJ 1.46r (http://imagej.nih.gov/ij). The outlines of the ROIs (cerebral cortex, thalamus, and hippocampus) were manually delineated in each section. Anterior delineation in the cerebral cortex was a line connecting the rhinal fissure and anterior tip of the external capsule. Dorsally it was delineated by the external surface of the brain, ventrally by the external capsule, and at posterior level by the dorsal subiculum. The anterior, ventral, and dorsal boundaries of the thalamus were defined by the surrounding white matter in the fimbria, internal capsule and cerebral peduncle and the hippocampus, while the posterior boundary was approximated by connecting the brachium of the superior colliculus and cerebral peduncle with a curved line encompassing geniculate nucleus. This delineation may also include the subthalamic nuclei. The delineation of the hippocampus included the three cornu ammonis subfields (CA1, CA2 and CA3), the dentate gyrus, and subiculum, and was at anterior level defined against the fimbria, dorsally and posteriorly against the external capsule, and ventrally against the thalamus. Images were binarized by selecting a threshold value in ImageJ, which yielded boundary definitions best corresponding to the observed plaque boundaries. The same threshold value was used for all sections. The area of each ROI and the area of labeled objects within each ROI were calculated, and the area fraction in percent (labeled area/ ROI area ×100) was used as a measure of plaque load. For each animal the mean area fractions of three sections were used for the final analysis.

### Statistical analysis

For immunohistochemistry, ANOVA assay and paired t-test in InStat (GraphPad Softwear, San Diego, CA, USA) were used to analyze amyloid plaque deposition in different groups (control vs. treated). For hormone analysis, Mann Whitney test and two-tailed unpaired t-test in InStat (GraphPad Softwear, San Diego, CA, USA) were used. A p-value less than 0.05 were considered a statistically significant difference.

For gene expression, the log2 transformed fold change values (2^−ΔΔCt^) were used for statistical analysis by applying JMP 10.0 software (SAS Institute Inc, Cary, NC, USA). Differences in gene expression were evaluated by Wilcoxon signed rank test.

## Results

### Hormone analysis

Blood samples showed significant lower levels of E2 when the Leuprorelin acetate treated animals were compared with the vehicle treated animals (p<0.001). The same results were found when males and females were investigated separately (p<0.01 for males treated with Gnrh-a versus vehicle and p<0.01 for females treated with Gnrh-a compared to vehicle).

**Figure 1:**
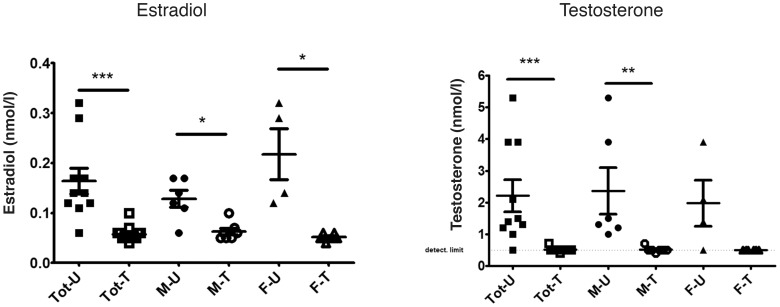
Estradiol (E2) and testosterone analyses in serum. Hormonal analyses of estradiol (E2) and testosterone show a decline in estradiol and testosterone levels both in males and females, after treatment with Leuprorelin acetate. Significant results are indicated with asterisk (**p<0.01, ***p<0.001). Tot-U - Total (females and males) untreated, Tot-T – Total treated, M-U – Males untreated, M-T – Males treated, F-U – Females untreated, F-T – Females treated.

The blood samples were also analyzed for testosterone levels. It showed that all treated animals had serum levels of testosterone less than 0.5 nmol/l (p<0.05). The results are presented in Figure 1.

### Gene expression

**Figure 2:**
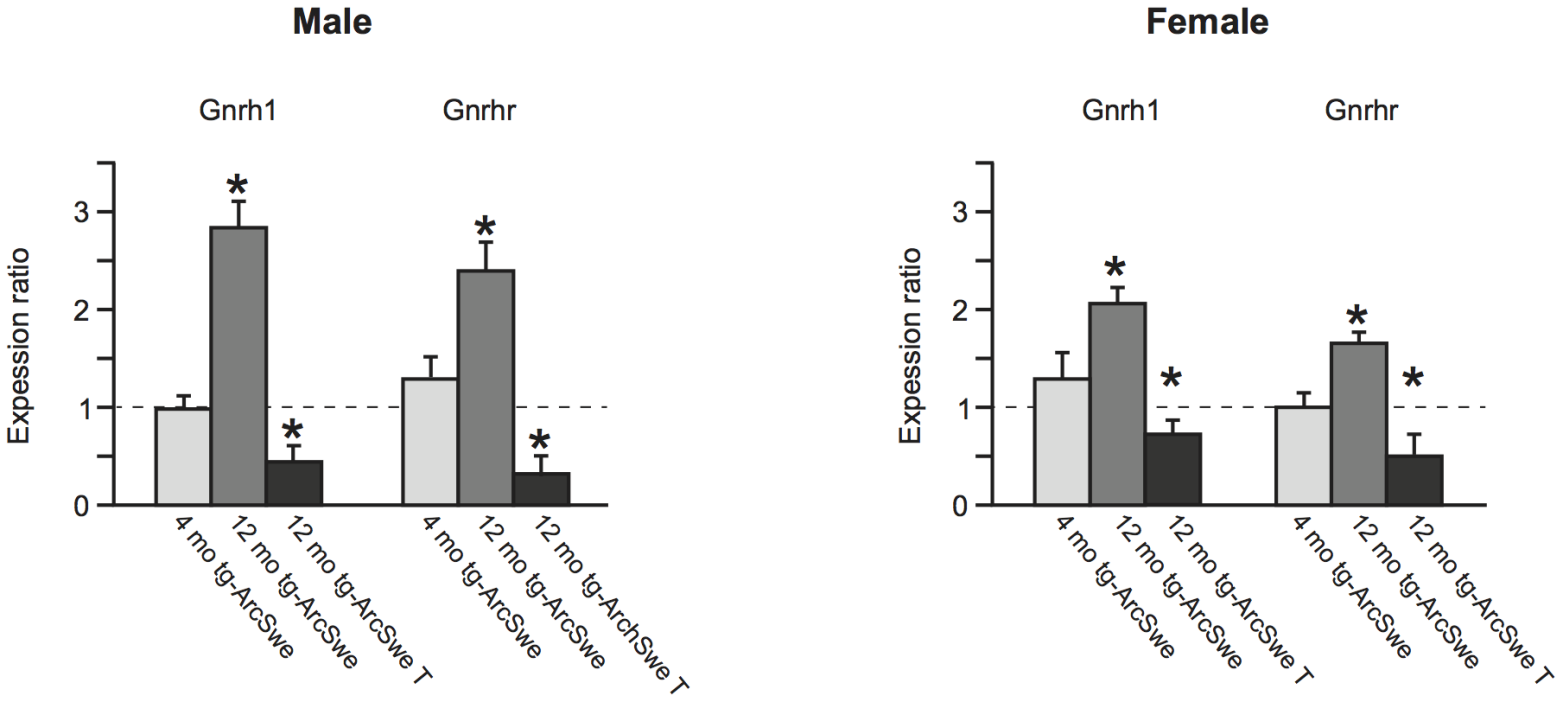
mRNA expression levels across the groups at 4 and 12 months. Hippocampal expression of Gnrh and Gnrhr transcripts in male and female tgArcSwe mice related to control groups. Data are represented as fold change (FC) and standard error mean (SEM). The fold change values are derived from relative quantification and normalization to the expression of two reference genes (B2m and Gusb). Genes with values higher than 1 indicate increased mRNA expression, values lower than 1 decreased mRNA expression in target group relative to control group. Light grey bars demonstrate the fold change value of gene expression in 4 months old tgArcSwe relative to age-matched WT-mice; Grey bars demonstrate the fold change value of gene expression level in 12 months old tgArcSwe relative to age-matched WT-mice. Black bars demonstrate the fold change values of gene expression in 12 months old Gnrh-a treated tgArcSwe relative to vehicle treated tgArcSwe. Both sexes are presented separately. Significant (Wilcoxon signed rank test, p<0.05) changes in gene expression are indicated by asterisks.

To investigate the association between *Gnrh/Gnrhr* and AD we assessed gene expression levels in hippocampus of tgArcSwe mice at 4 months and 12 months. In addition, we analyzed gene expression level at 12 months after 8 months of treatment with Leuprorelin acetate (25 ng/g). The results are presented in Figure 2. Our gene expression analyses show that at 12 months of age, both *Gnrh* (p<0.001 in both sexes) and *Gnrhr* (p<0.01 male and p<0.001 female) mRNA expression were significantly elevated in tgArcSwe compared to age-matched WT in both sexes. Furthermore, gene expression analysis of 4 months old WT mice relative to 12 months old WT mice revealed elevated expression of *Gnrh* (p<0.05 for male and female) and *Gnrhr* (p<0.05 for male and female). Strikingly, after 8 months of treatment with Leuprorelin acetate, gene expression of both *Gnrh* (p<0.001 male and p<0.01 female) and *Gnrhr* (p<0.001 in both sexes) were down-regulated when Leuprorelin acetate-treated tgArcSwe to vehicle-treated tgArcSwe at the same age were compared. At 4 months no significant differences in *Gnrh/Gnrhr* were observed. All fold change values, standard errors and p-values are presented in Table 3. The mRNA expression of *AβPP* was significantly elevated at 4 months in both sexes, consistent with previous findings [27].

**Table 3:** Gene expression results across different groups, at different age.

			Gnrh			Gnrhr		
	Age (months)	Sex	FC	SEM	p-value	FC	SEM	p-value
TgArcSwe-mice relative to wild-type mice	4	M	0.98	0.14	0.65	1.30	0.23	0.57
	4	F	1.23	0.26	0.54	0.98	0.11	0.46
	12	M	2.89	0.23	<0.001	2.43	0.28	<0.01
	12	F	2.00	0.11	<0.001	1.60	0.10	<0.001
Treated relative to untreated tgArcSwe-mice	12	M	0.46	0.14	<0.001	0.33	0.15	<0.001
	12	F	0.67	0.15	<0.01	0.51	0.19	<0.001
4 months wild-type mice relative to 12 months wild-type mice	4/12	M	2.56	0.18	<0.05	3.21	0.23	<0.05
	4/12	F	2.33	0.13	<0.05	3.56	0.12	<0.05

T =  treatment with Leuprorelin acetate, VT =  vehicle treated, FC =  fold change, SEM =  standard error mean.

### Immunohistochemistry

**Figure 3:**
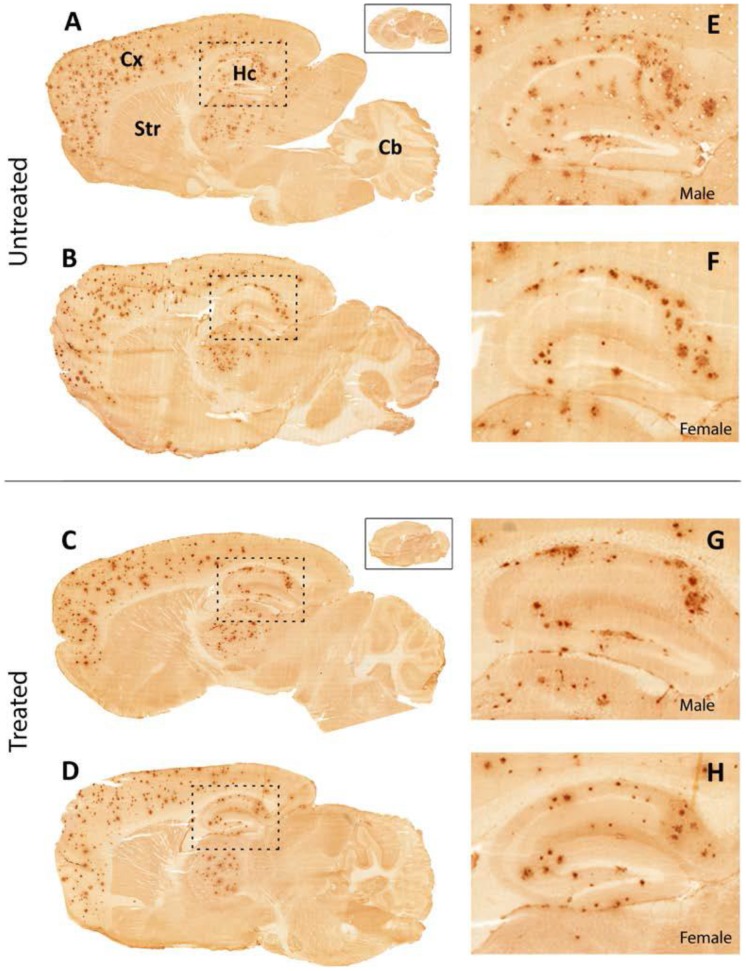
Immunohistochemistry for Aβ-deposits in the male and female mouse brain. Immunohistochemistry for amyloid (Aβx-40) in the right hemisphere of male and female mouse brain. Aβ-deposits are found throughout the cerebral cortex (Cx) and hippocampus (Hc) in all animals regardless of treatment. Enlarged images E–H show morphological details in the hippocampus. Insets show sections from wild-type animals of corresponding genders demonstrating that background staining was low.

**Figure 4:**
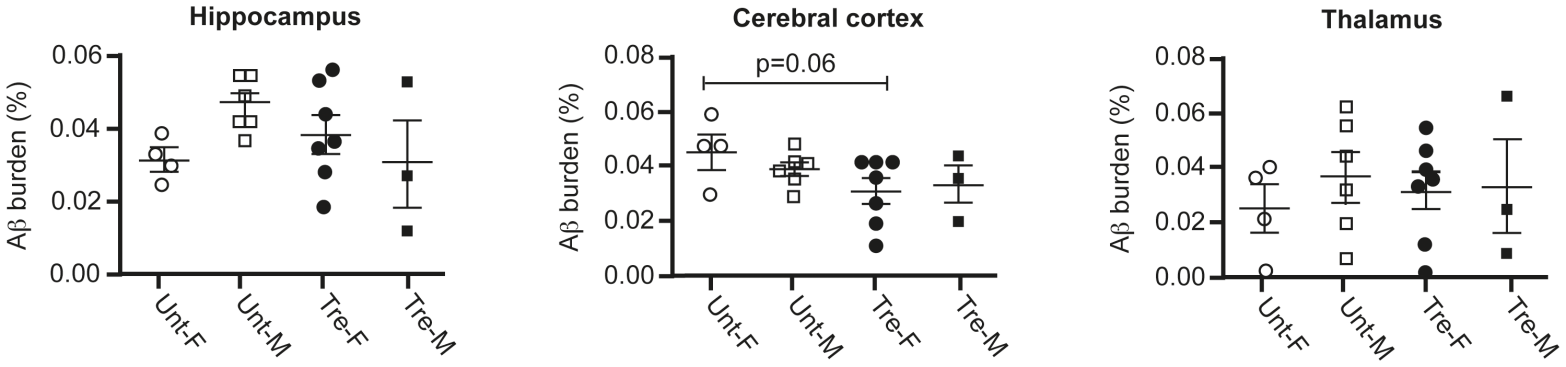
Effect of Gnrh-analog treatment on Aβ-deposits across different regions of the brain. Aβ-deposition in the cerebral cortex, hippocampus and thalamus of the treated (tre-) and vehicle-treated (unt-) tgArcSwe mice. For each animal the mean area fractions of three sections were used for the final analysis. Results are presented with means and standard error means (SEM). Unt-untreated females (○) and males (□), Tre-treated females (•) and males (▪). There was no significant change between the groups, but a trend towards lower plaque deposition in treated female cerebral cortex (p = 0.06) compared to vehicle treated controls.

The amount of Aβx-40 labeling was determined by quantitative image analysis. Depositions of amyloid-beta (Aβ) loads in cerebral cortex, hippocampus and thalamus of 12 months old tgArcSwe mice are shown in Figure 3. The average plaque load in the hippocampus of 12 months old female and male tgArcSwe mice receiving Leuprorelin acetate (7 female and 3 male mice), did not differ significantly when compared to untreated sex- and age-matched tgArcSwe mice (4 female and 6 male mice), (Figure 4). However, we found a trend indicating a lower plaque load in the cerebral cortex of drug treated females (p = 0.06) compared to vehicle-treated females. Comparisons of plaque load in cerebral cortex and thalamus in the same animal groups did not reveal any differences. Our conclusions are mostly based on the female group as the group of treated male mice was reduced due to an inadvertent loss of animals, although the results among the male mice pointed in the same direction.

## Discussion

This study provides the first evidence that *Gnrh* and *Gnrhr* mRNAs are significantly upregulated in 12 months old tgArcSwe mice compared to nontransgenic mice. We further demonstrate that treatment with Leuprorelin acetate leads to lower levels of E2 and testosterone in serum, as well as an effective down-regulation of *Gnrh* and *Gnrhr* mRNA expression in these mice. Interestingly, at young age (4 months old animals) there was no significant differences in *Gnrh* and *Gnrhr* mRNA expression between transgenic and WT-mice (prior to treatment and onset of plaque deposition).

It has been reported that the hippocampal Gnrh system is acutely sensitive to both age and reproductive status, as Gnrhr expression is increasing in ageing rats [34]. This result is in concordance with our data, showing increased mRNA expression of *Gnrh* and *Gnrhr* in 12 months WT compared to 4 months WT. Our findings of strongly increased *Gnrh/Gnrhr* expression in 12 months old AD-mice relative to age-matched WT show that *Gnrh/Gnrhr* expression increases even further in association with amyloid pathology. This is in line with the idea that Gnrh/Gnrhr contributes to the development of AD pathology and with studies reporting that Gnrh/Gnrhr influences hippocampal synaptic activity and impacts central nervous system physiology as well as pathophysiology [18], [35]. However, it remains to be resolved whether this is a direct influence of Aβ-pathology or a consequence of secondary pathology, e.g. micro-/astrogliosis with release of substances that increase Gnrh/Gnrhr expression in hippocampus.

The potential of Gnrh-a treatment was first demonstrated in a mouse study in which intervention lowered hippocampal plaque load and prevented AD-related cognitive dysfunctions [36], [37]. Additionally, it has been reported that Gnrh-a treatment reduced Aβ concentration in total brain after 2 months of treatment in C57BL/6 mice [36]. This finding suggests that Gnrh-a treatment decreases Aβ levels by suppressing serum gonadotropins. Further, luteinizing hormone (LH) promoted AβPP-processing towards the amyloidogenic pathway in a neuroblastoma cell line, as evidenced by increased Aβ-formation and secretion [36]. Another study, in which very old mice (tg2576, carrying the Swedish AβPP mutation) were treated with a Gnrh-a for two months, resulted in decreased hippocampal Aβ-deposition and improved cognitive functions among the aged transgenic mice [37]. Despite these positive reports, there have also been several subtle yet significant negative data regarding the effect of Gnrh-a on cognitive functions [38]–[40]. This needs to be considered when hormone therapy is given to prostate cancer patients. Because individuals with AD show an increase in circulating gonadotropins (LH and FSH) compared with age-matched controls, gonadotropins have received increased attention over the last years [41]–[44]. Mechanistically, Gnrh has been suggested to promote the reactivation of mitotic signaling pathways that occurs early in AD pathogenesis. Although LH might mediate these effects, it is also possible that Gnrh-a treatment mediates its effects directly. It is known that Gnrhr signaling system differs in a cell specific manner, but it is not finally concluded which specific downstream signaling pathway is regulated by Gnrh-a treatment in the different region of the brain such as hippocampus and pituitary. Possibly, these variations could be due to variation in the Gnrhr protein sequences or post translational splicing [29], [45].

Based on these findings, we expected that Leuprorelin acetate would modulate *Gnrh* and *Gnrhr* expression in tgArcSwe, and also exert an impact on amyloid plaque load in the animals. However, we did not record any significant differences in plaque load between groups after 8 months of treatment, although we observed a trend towards a decreased cortical plaque load in treated female animals compared to vehicle-treated controls. We based our conclusions mostly on the female group, as the group of treated male mice was reduced due to an inadvertent loss of animals. However, the male qPCR data points in the same direction as the female. Our recent study of plaque load variability in tgArcSwe mice [26] indicates that it would have been possible to detect a major change in plaque load, while smaller changes might have been missed due to insufficient sample size. The fact that tgArcSwe is a stronger genetically driven model than e.g. tg2576, might also explain differences in therapeutic effects. The modifying effect of Leuprorelin acetate could be relatively weaker than the (trans)-genetic effect in tgArcSwe and thereby difficult to detect.

In this study we used an Aβx-40 antibody to assess amyloid burden. It has previously been demonstrated that Aβ antibodies stain the same, homogeneous population of cored Aβ-plaques in the tgArcSwe model, irrespective of whether the antibodies recognize the N- or C-terminal epitopes in Aβ. Therefore in tgArcSwe mice the 6E10, Aβx-40 or Aβx-42 antibodies will produce similar labeling pattern and indicate the same Aβ burden, at least at the light-microscopic level. This probably reflects the fact that the Arctic AβPP mutation makes Aβ-peptides far more prone to aggregate, including Aβ1-40 [26], [27], [32].

We designed our project to evaluate the effect of Leuprorelin acetate on plaque deposition in a well described mouse model of Alzheimer's disease. The present gene expression analyses were restricted to a single hormone and its receptor only. Obviously, this confers a limitation on the conclusions that can be made regarding the hormonal processes that affect the development of AD. The present findings warrant further studies using microarray gene expression profiling along with proteomics analyses.

In conclusion, *Gnrh* and its receptor are overexpressed in 12 months old tgArcSwe compared to WT. Leuprorelin acetate treatment was shown to affect the expression of the gene encoding the hormone receptor as well as the gene encoding the hormone itself, consistent with down-regulation of endogenous endocrine systems. The present study brings to the fore the involvement of hormonal changes in AD and the prospect of mitigating these through targeted treatment.

## References

[1] Wimo A, Jönsson L, Bond J, Prince M, Winblad B (2013) The worldwide economic impact of dementia 2010. Alzheimers Dement 9: 1–11. 23305821 10.1016/j.jalz.2012.11.006

[2] Wimo A, Winblad B, Jönsson L (2007) An estimate of the total worldwide societal costs of dementia in 2005. Alzheimers Dement 3: 81–91. 19595921 10.1016/j.jalz.2007.02.001

[3] Francis PT, Parsons CG, Jones RW (2012) Rationale for combining glutamatergic and cholinergic approaches in the symptomatic treatment of Alzheimer's disease. Expert Rev Neurotherapeutics 12: 1351–1365. 10.1586/ern.12.124 23234396

[4] Selkoe DJ (1997) Alzheimer's disease: genotypes, phenotype, and treatments. Science 275: 630–631. 9019820 10.1126/science.275.5300.630

[5] Verri M, Pastoris O, Dossena M, Aquilani R, Guerriero F, et al (2012) Mitochondrial alterations, oxidative stress and neuroinflammation in Alzheimer's disease. Int J Immunopath Ph 25: 345–353. 10.1177/039463201202500204 22697066

[6] Bowen RL (2001) Sex hormones, amyloid protein, and Alzheimer disease. JAMA 286: 790–791. 11497529 10.1001/jama.286.7.790

[7] Hogervorst E, Bandelow S, Combrinck M, Smith AD (2004) Low free testosterone is an independent risk factor for Alzheimer's disease. Exp Gerontol 39: 1633–1639. 15582279 10.1016/j.exger.2004.06.019

[8] Zhang G, Li J, Purkayastha S, Tang Y, Zhang H, et al (2013) Hypothalamic programming of systemic ageing involving IKK-β, NF-κB and GnRH. Nature 497: 211–216. 23636330 10.1038/nature12143 PMC3756938

[9] D'Amico AV, Braccioforte MH, Moran BJ, Chen MH (2010) Luteinizing-hormone releasing hormone therapy and the risk of death from Alzheimer Disease. Alzheimer Dis Assoc Disord 24: 84–89. 10.1097/wad.0b013e31819cb8f4 20556875

[10] Nuruddin S, Wojniusz S, Ropstad E, Krogenæs A, Evans NP, et al (2013) Peri-pubertal gonadotropin-releasing hormone analog treatment affects hippocampus gene expression without changing spatial orientation in young sheep. Behav Brain Res 242: 9–16. 23266521 10.1016/j.bbr.2012.12.027

[11] Nuruddin S, Krogenæs A, Brynildsrud OB, Verhaegen S, Evans NP, et al. (2013) Peri-pubertal gonadotropin-releasing hormone agonist treatment affects sex biased gene expression of amygdala in sheep. Psychoneuroendocrinology. In press 10.1016/j.psyneuen.2013.09.011 24103890

[12] Skinner DC, Albertson AJ, Navratil A, Smith A, Mignot M, et al (2009) Effects of gonadotrophin-releasing hormone outside the hypothalamic-pituitary-reproductive axis. J Neuroendocrinol 21: 282–292. 19187469 10.1111/j.1365-2826.2009.01842.x PMC2669307

[13] He D, Funabashi T, Sano A, Uemura T, Minaguchi H, et al (1999) Effects of glucose and related substrates on the recovery of the electrical activity of gonadotropin-releasing hormone pulse generator which is decreased by insulin-induced hypoglycemia in the estrogen-primed ovariectomized rat. Brain Research 820: 71–76. 10023032 10.1016/s0006-8993(98)01358-4

[14] Lu F, Yang JM, Wu JN, Chen YC, Kao YH, et al (1999) Activation of gonadotropin-releasing hormone receptors produces neuronal excitation in the rat hippocampus. Chin J Physiol 42: 67–71. 10513601

[15] Gault PM, Maudsley S, Lincoln GA (2003) Evidence that gonadotropin-releasing hormone II is not a physiological regulator of gonadotropin secretion in mammals. J Neuroendocrinol 15: 831–839. 12899677 10.1046/j.1365-2826.2003.01065.x

[16] Millar RP (2005) GnRHs and GnRH receptors. Anim Reprod Sci 88: 5–28. 16140177 10.1016/j.anireprosci.2005.05.032

[17] Stevenson TJ, Hahn TP, MacDougall-Shackleton SA, Ball GF (2012) Gonadotropin-releasing hormone plasticity: A comparative perspective. Front Neuroendocrin 33: 287–300. 10.1016/j.yfrne.2012.09.001 PMC3484179 23041619

[18] Wang L, Chadwick W, Park S, Zhou Y, Silver N, et al (2010) Gonadotropin-releasing hormone receptor system: modulatory role in aging and neurodegeneration. Cns Neurol Disord-DR 9: 651–660. 10.2174/187152710793361559 PMC2967575 20632963

[19] Bryan KJ, Mudd JC, Richardson SL, Chang J, Lee HG, et al (2010) Down-regulation of serum gonadotropins is as effective as estrogen replacement at improving menopause-associated cognitive deficits. J Neurochem 112: 870–881. 19943850 10.1111/j.1471-4159.2009.06502.x PMC2886127

[20] Evans NP, Robinson JE, Erhard HW, Ropstad E, Fleming LM, et al (2012) Development of psychophysiological motoric reactivity is influenced by peripubertal pharmacological inhibition of gonadotropin releasing hormone action-Results of an ovine model. Psychoneuroendocrinology 37: 1876–1884. 22534405 10.1016/j.psyneuen.2012.03.020

[21] Wojniusz S, Vögele C, Ropstad E, Evans N, Robinson J, et al (2011) Prepubertal gonadotropin-releasing hormone analog leads to exaggerated behavioral and emotional sex differences in sheep. Horm Behav 59: 22–27. 20934426 10.1016/j.yhbeh.2010.09.010

[22] Grigorova M, Sherwin BB, Tulandi T (2006) Effects of treatment with leuprolide acetate depot on working memory and executive functions in young premenopausal women. Psychoneuroendocrinology 31: 935–947. 16831518 10.1016/j.psyneuen.2006.05.004

[23] Nelson CJ, Lee JS, Gamboa MC, Roth AJ (2008) Cognitive effects of hormone therapy in men with prostate cancer. Cancer 113: 1097–1106. 18666210 10.1002/cncr.23658 PMC4333639

[24] Simpkins JW, Perez E, Wang X, Yang S, Wen Y, et al (2009) Review: The potential for estrogens in preventing Alzheimer's disease and vascular dementia. Ther Adv Neurol Disord 2: 31–49. 19890493 10.1177/1756285608100427 PMC2771945

[25] Wilson AC, Salamat MS, Haasl RJ, Roche KM, Karande A, et al (2006) Human neurons express type I GnRH receptor and respond to GnRH I by increasing luteinizing hormone expression. J Endocrinol 191: 651–663. 17170222 10.1677/joe.1.07047

[26] Lillehaug S, Syverstad GH, Nilsson LNG, Bjaalie JG, Leergaard TB, et al (2014) Brainwide distribution and variance of amyloid-beta deposits in tg-ArcSwe mice. Neurobiol Aging 35: 556–564. 24126157 10.1016/j.neurobiolaging.2013.09.013

[27] Lord A, Kalimo H, Eckman C, Zhang XQ, Lannfelt L, et al (2006) The Arctic Alzheimer mutation facilitates early intraneuronal Aβ aggregation and senile plaque formation in transgenic mice. Neurobiol Aging 27: 67–77. 16298242 10.1016/j.neurobiolaging.2004.12.007

[28] AV Schally, AM Comaru-Schally, A Nagy, M Kovacs, K Szepeshazi, et al (2001) Hypothalamic hormones and cancer. Front Neuroendocrinol 22: 248–291. 11587553 10.1006/frne.2001.0217

[29] Ehlers K, Halvorson L (2013) Gonadotropin- releasing hormone (GnRH) and GnRH Receptors (GnRHR). Glob libr. Women's med., ISSN: 1756–2228

[30] Vandesompele J, De Preter K, Pattyn F, Poppe B, Van Roy N, et al (2002) Accurate normalization of real-time quantitative RT-PCR data by geometric averaging of multiple internal control genes. Genome Biol 3: research0034.1–research0034.11. 12184808 10.1186/gb-2002-3-7-research0034 PMC126239

[31] Untergasser A, Nijveen H, Rao X, Bisseling T, Geurts R, et al (2007) Primer3Plus, an enhanced web interface to Primer3. Nucleic Acids Res 35: W71–74. 17485472 10.1093/nar/gkm306 PMC1933133

[32] Näslund J, Haroutunian V, Mohs R (2000) Correlation between elevated levels of amyloid β-peptide in the brain and cognitive decline. JAMA 283: 1571–1577. 10735393 10.1001/jama.283.12.1571

[33] Sedgewick J (2008) Scientific Imaging with Photoshop: Methods, Measurement, and Output. Peachpit Press, California. ISBN-10: 0321514335.

[34] Badr M, Marchetti B, Pelletier G (1988) Modulation of hippocampal LHRH receptors by sex steroids in the rat. Peptides 9: 441–442. 2836829 10.1016/0196-9781(88)90283-5

[35] Meethal S, Smith M, Bowen R, Atwood C (2005) The gonadotropin connection in Alzheimer's disease. Endocrine 26: 317–325. 16034187 10.1385/ENDO:26:3:317

[36] Bowen RL, Verdile G, Liu T, Parlow AF, Perry G, et al (2004) Luteinizing Hormone, a reproductive regulator that modulates the processing of amyloid-beta precursor protein and amyloid-beta deposition. J Biol Chem 279: 20539–20545. 14871891 10.1074/jbc.M311993200

[37] Casadesus G, Webber KM, Atwood CS, Pappolla MA, Perry G, et al (2006) Luteinizing hormone modulates cognition and amyloid-beta deposition in Alzheimer APP transgenic mice. BBA-Mol Basis Dis 1762: 447–452. 10.1016/j.bbadis.2006.01.008 16503402

[38] Green HJ, Pakenham KI, Headley BC, Yaxley J, Nicol DL, et al (2002) Altered cognitive function in men treated for prostate cancer with luteinizing hormone-releasing hormone analogues and cyproterone acetate: a randomized controlled trial. BJU Int 90: 427–432. 12175403 10.1046/j.1464-410x.2002.02917.x

[39] Cherrier MM, Rose AL, Higano C (2003) The effects of combined androgen blockade on cognitive function during the first cycle of intermittent androgen suppression in patients with prostate cancer. J Urol 170: 1808–1811. 14532781 10.1097/01.ju.0000091640.59812.83

[40] Sherwin BB (2003) Steroid hormones and cognitive functioning in aging men: a mini-review. J Mol Neurosci 20: 385–393. 14501023 10.1385/JMN:20:3:385

[41] Marlatt MW, Webber KM, Moreira PI, Lee HG, Casadesus G, et al (2005) Therapeutic opportunities in Alzheimer disease: One for all or all for one? Curr Med Chem 12: 1137–1147. 15892629 10.2174/0929867053764644

[42] Verdile G, Laws SM, Henley D, Ames D, Bush AI, et al (2014) Associations between gonadotropins, testosterone and β amyloid in men at risk of Alzheimer's disease. Mol Psychiatry 19: 69–75. 23089633 10.1038/mp.2012.147

[43] Rodrigues MA, Verdile G, Foster JK, Hogervorst E, Joesbury K, et al (2008) Gonadotropins and Cognition in Older Women. J Alzheimers Dis 13: 267–274. 18430994 10.3233/jad-2008-13304

[44] Barron A, Verdile G, Martins R (2006) The role of gonadotropins in Alzheimer's disease. Endocrine 29: 257–269. 16785601 10.1385/ENDO:29:2:257

[45] Nogami H, Hoshino R, Ogasawara K, Miyamoto S, Hisano S (2007) Region-specific expression and hormonal regulation of the first exon variants of rat prolactin receptor mRNA in rat brain and anterior pituitary gland. J Neuroendocrinol 19: 583–93. 17620100 10.1111/j.1365-2826.2007.01565.x

